# Guided Self-Help Works: Randomized Waitlist Controlled Trial of Pacifica, a Mobile App Integrating Cognitive Behavioral Therapy and Mindfulness for Stress, Anxiety, and Depression

**DOI:** 10.2196/12556

**Published:** 2019-06-08

**Authors:** Christine Moberg, Andrea Niles, Dale Beermann

**Affiliations:** 1 Pacifica Labs, Inc Minneapolis, MN United States; 2 University of California-San Francisco San Francisco, CA United States

**Keywords:** mHealth, anxiety, depression, stress, cognitive behavioral therapy, smartphone app

## Abstract

**Background:**

Despite substantial improvements in technology and the increased demand for technology-enabled behavioral health tools among consumers, little progress has been made in easing the burden of mental illness. This may be because of the inherent challenges of conducting traditional clinical trials in a rapidly evolving technology landscape.

**Objective:**

This study sought to validate the effectiveness of Pacifica, a popular commercially available app for the self-management of mild-to-moderate stress, anxiety, and depression.

**Methods:**

A total of 500 adults with mild-to-moderate anxiety or depression were recruited from in-app onboarding to participate in a randomized waitlist controlled trial of Pacifica. We conducted an all-virtual study, recruiting, screening, and randomizing participants through a Web-based participant portal. Study participants used the app for 1 month, with no level of use required, closely mimicking real-world app usage. Participants in the waitlist group were given access to the app after 1 month. Measurements included self-reported symptoms of stress, anxiety, depression, and self-efficacy. We performed an intent-to-treat analysis to examine the interactive effects of time and condition.

**Results:**

We found significant interactions between time and group. Participants in the active condition demonstrated significantly greater decreases in depression, anxiety, and stress and increases in self-efficacy. Although we did not find a relationship between overall engagement with the app and symptom improvement, participants who completed relatively more thought record exercises sustained improvements in their symptoms through the 2-month follow-up to a greater degree than those who completed fewer. In addition, we found that participants who reported concomitantly taking psychiatric medications during the trial benefitted less from the app, as measured by the symptoms of anxiety and stress.

**Conclusions:**

This study provides evidence that Pacifica, a popular commercially available self-help app, is effective in reducing self-reported symptoms of depression, anxiety, and stress, particularly among individuals who utilize thought records and are not taking psychiatric medication.

**Trial Registration:**

ClinicalTrials.gov NCT03333707; https://clinicaltrials.gov/ct2/show/NCT03333707 (Archived by WebCite at http://www.webcitation.org/78YE07ADB)

## Introduction

### Background

In the United States, in a given year, over 19% of adults experience an anxiety disorder [1] and 6.7% experience a major depressive episode [2]. Decades of mental health care research have resulted in the development of effective nonpharmacological therapies for anxiety and depression, one of which is cognitive behavioral therapy (CBT) [3,4]. CBT is among the best-researched nonpharmacological treatments for depression and anxiety [5,6]. However, traditional CBT requires a well-trained practitioner and significant time commitment both inside and outside of the therapy room from the client; dissemination of this treatment has been somewhat limited despite its demonstrated efficacy [7].

Untreated mental illness has well-documented negative effects on physical health, health care costs, productivity, and well-being [8-10], and mental health and substance use disorders are the leading causes of disease burden in the United States [11]. Unfortunately, only 43% of people with mental illness in the United States receive professional care because of difficulties with access to care, stigma, cost, or time involved in seeking treatment [2].

Technology-enabled interventions hold great promise to ease some of the burden of mental illness due to their ability to scale and reduce barriers to entry such as cost and stigma. In fact, computerized or internet-based implementations of CBT have existed for years and many have been demonstrated to be effective for a number of mental health conditions [12-15]. However, although these products exist, they have made little impact on reducing the overall disease burden and implementation has lagged behind development [16].

Similar to the research-practice gap that exists for much of mental health treatment, digital tools that perform well in closely monitored, tightly controlled research settings do not always translate into widely utilized programs among consumers. This may be because of poor usability and design or concerns about privacy and data security [17,18]; in some cases, these apps are never made available to the public. More real-world studies that demonstrate the effectiveness of mobile-delivered treatment programs are needed.

User retention and engagement are critical to the success of digital health interventions [17,19]. This is no different than in traditional or in-person psychotherapy, where treatment retention and engagement has long been a topic of study [20]. LeBeau et al [21] defined treatment engagement as homework compliance and found that greater compliance predicted better outcomes among patients receiving CBT for anxiety disorders. However, with digital tools, engagement has often been defined by the number of uses or logins. The relationship between logins or time spent using the app or digital tool is somewhat complex; it is not always the case that greater use correlates with greater symptom improvement. For example, 1 study found that increased usage of a website for depression treatment corresponded with less symptom improvement, and the authors suggest the explanation that individuals who derived benefit from the treatment discontinued their use early [22]. Kelders et al [23] examined digital treatment adherence and identified that engagement-related analyses should consider the types of actions taken by participants (eg, didactic lessons vs feedback vs skills practice) and the time at which the individual became nonadherent (ie, early vs late). Tying engagement to outcomes can also be complex. van Gemert-Pijnen et al [24] examined participants’ number of logins and use of various platform features and found a somewhat complex relationship between logins, feature use, and impact on depressive symptoms.

There is also a rich literature on predictors of treatment response in CBT, both in-person and when administered via digital tools, though it is hard to draw any clear conclusions about for whom CBT does or does not work. Høifødt et al [25] found contributions from both some demographic and clinical factors in a Web-based CBT intervention for depression. Specifically, having had more depressive episodes, being married or cohabitating, and having higher life satisfaction predicted better response. Myrh et al [26] reviewed the literature on predictors of treatment response to in-person CBT and described that although several studies have found little-to-no strong predictors of response to treatment, others have found that higher socioeconomic status and being married predict better outcomes. Dryman et al [27] conducted a study of the Internet-based CBT (ICBT) mobile app, Joyable, to examine its efficacy for treating social anxiety and found that responders (vs nonresponders) had lower baseline symptoms and spent more days in the program. Responders also called their coaches more and completed more exposures. The groups did not differ on age or gender. MoodHacker is a CBT-based app for individuals with depression. In a randomized controlled trial (RCT) examining the efficacy of MoodHacker, Birney et al [28] found that depression symptom improvements were larger among study participants who had access to an employee assistance program (EAP), though the authors note that EAP access could actually represent more than just access to an in-person counselor (eg, non-EAP participants may have different motivations than those who encountered the study through EAPs). Given these mixed data, we were also interested in examining for whom the Pacifica app would be most effective.

### Objectives

Though it is not the first all-virtual RCT of a mental health support app [29,30], this study was an effort to advance the literature on the real-world use of technology-enabled CBT. We sought to test the effectiveness of Pacifica [31], a popular (over 2.4 million registered users at the time of submission) commercially available app for the self-management of stress, anxiety, and depression, in a sample of individuals with mild-to-moderate anxiety and depression who were seeking digital tools to address their mental health. We predicted that access to the app would help improve their symptoms and increase self-efficacy. In addition, we hypothesized that greater app usage, that is, more engagement, would result in larger symptom improvements. In addition, given the ongoing debate about active ingredients in therapy [32] and the transtheoretical approach of the app, we aimed to examine whether symptom improvement was related to the specific tools that were utilized (eg, thought records, meditation, and social posts). Finally, we evaluated whether demographic variables or baseline clinical features would affect the efficacy of our app-based intervention.

## Methods

### Study Design

This RCT compared an immediate intervention group (*Pacifica*) with a waitlist control group (*WL*). Both groups had access to treatment as usual at their discretion. The WL group was provided access to the intervention at the end of the 1-month waiting period. The immediate intervention group was re-assessed 2 months after their completion of the 1-month intervention period to examine the stability of symptom change. The study was registered with ClinicalTrials.gov (NCT03333707) and was approved by Salus IRB (Austin, TX).

### Recruitment, Screening, and Consent

We recruited participants for 3 months, from November 2017 through February 2018, via social media advertisements, a listing on ClinicalTrials.gov, and through an opt-in screen in the commercially available app itself. The vast majority of participants were recruited through the app itself. Pacifica does not advertise to acquire users, and most individuals find the app through app store searches, using terms such as *anxiety* or *stress* or via word of mouth.

We designed and built a Participant Portal that provided individuals with a *self*-*service* platform for screening, consent, and randomization to the app. To reduce duplicate enrollments, potential participants provided their telephone number and entered a code that was sent to them via short messaging service (SMS) text messaging. A prescreen required participants to be over the age of 18 years, fluent in English, with regular access to a smartphone, and no previous experience with Pacifica. After this prescreen, participants were provided with an informed consent document in an embedded PDF. A 4-question knowledge check confirmed that they had read and understood the document. To consent, participants entered their email address and password. Following consent, participants were screened to confirm eligibility. The Participant Portal is Web (not app) based, and therefore, participants could access the portal through any device with a Web browser. Participants assigned to the waitlist condition would not have been able to download and use the app (outside of the study) unless they signed up with a different email address than whichever address they used for this prescreen.

In addition to the criteria in the prescreen described above, the inclusion criterion was a score between 5 and 14 on the Generalized Anxiety Disorder 7-item (GAD-7) scale [33] or between 5 and 14 on the Patient Health Questionnaire 8-item (PHQ-8) scale [34]. Exclusion criteria were (1) score below 5 on GAD-7 and PHQ-8 or above 14 on either, (2) positive response on screener for previous diagnosis of bipolar disorder, schizophrenia, other psychotic spectrum disorder, or organic brain disease, and (3) currently pregnant. Given the all-virtual nature of the study, these criteria were selected to match the intensity of the intervention to the severity of the users, allowing us to balance participant safety with external validity and value of the data.

**Figure 1:**
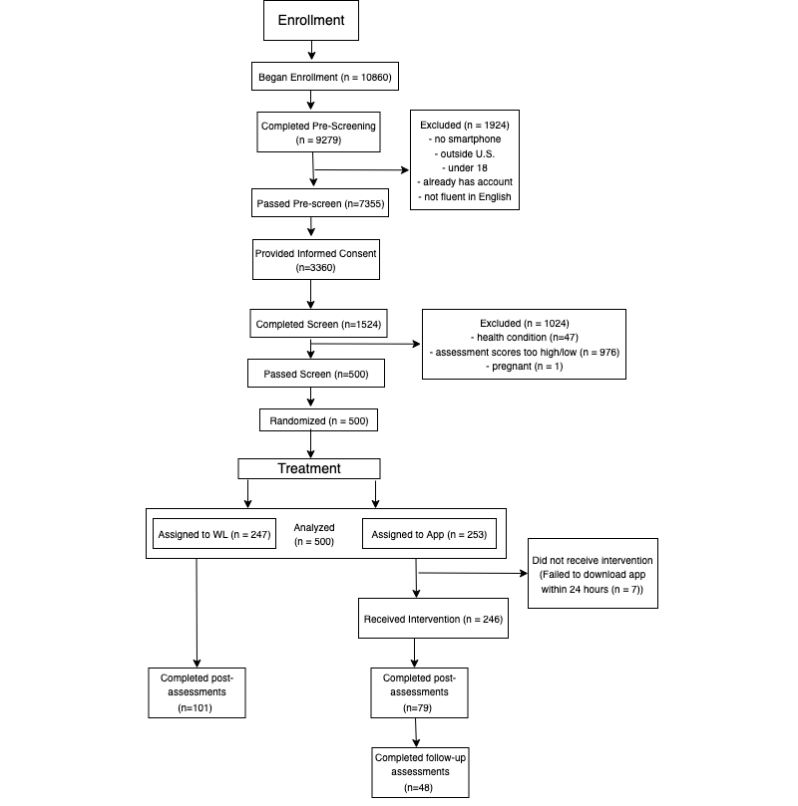
Consolidated Standards of Reporting Trials diagram.

An initial group of 9279 individuals completed the prescreen, 1524 completed informed consent, and 500 were randomized to groups. In addition, 7 individuals’ participation was discontinued for failing to download the app within 48 hours of screening and randomization. Of the 253 individuals who were assigned to the treatment group, 182 participants used Apple (iOS) devices and 61 used Android devices. Furthermore, 3 participants accessed Pacifica with both iOS and Android devices. Participants were assessed using questionnaire measures, the details for which are below. See the CONSORT flow diagram (Figure 1) for reasons for exclusion and experimental compliance.

### Intervention

Participants in the Pacifica group were given access to Pacifica Premium, versions 5.7 through 5.9.1. Updates to the app that occurred during the research study were bug fixes and performance improvements and would not have impacted the therapeutic approach or usability of the app. *Descriptions of the app below are as it was in the research study and may not be exactly the same as the currently available product.* Pacifica is a mobile app marketed as a guided self-help tool for the management of stress, anxiety, and depression. It is not described as a treatment for any particular diagnosis nor is it described as a substitute for professional treatment. At onboarding, users select up to 3 goals on which to work from a list of 8 options. The app prompts users once per day to rate their mood and, based on their mood rating, recommends activities to improve their mood via *Suggested Activities* (ie, ecological momentary intervention; see the studies by Schueller et al and Lovibond and Lovibond [35,36]). There are also 35 days of *Guided Paths* that are approximately 10-min audio psychoeducational lessons with paired activities. Alternatively, users can use the app *buffet style*, using whichever tools they find helpful, whenever they choose. Participants in the study were not provided guidance or suggestions regarding the amount of level of app usage beyond any prompts in the app (described above).

Descriptions and details on the different activities are given below:

#### Mood

Allows users to rate their mood on a scale from *Great* to *Awful*. Users may optionally label specific emotions or attach a journal-style note to their mood rating.

#### Health

Allows users to track health behaviors such as sleep, caffeine consumption, and exercise. Users can customize their health behaviors and goals for each health behavior (ie, how many hours of sleep or number of cups of coffee). They may optionally set up an alarm so that Pacifica sends them a notification to remind them to enter their health data.

#### Meditation/Relax

Offers over 40 audio activities, most of which are intended to promote mindfulness or relaxation. These include deep breathing, progressive muscle relaxation, and a variety of mindfulness activities.

#### Thoughts

Offers 9 different activities to help users re-examine their thoughts, identify cognitive distortions, and reframe thoughts. The prompts are divided into 3 groups: *basics*, which help the user recognize the relationship between their thoughts and emotions, learn about cognitive distortions, and reframe negative thoughts; *journal prompts*, which allow for free writing; and *advanced tools*. The advanced tools include one that creates a pie chart to help users identify other factors beyond themselves that may contribute to events, a tool that asks a series of questions to help them evaluate evidence, and an activity that guides them to drill down and identify core beliefs. All of these tools are guided exercises and do not provide automatic analysis of the individual’s text inputs.

#### Goals

Allows the user to create a list of challenges to complete to meet their longer-term goals. A fairly generic list tool, it can be customized to address anxiety by enabling the user to build and address a hierarchy of feared or avoided situations. Users coping with depression can use the list to engage in behavioral activation to re-engage with life. The tool prompts the user to rate the perceived difficulty of the item before and after engagement to help them recognize that their initial estimation of the challenge may not be accurate. There are example items that may be chosen or users can type in their own goals.

#### Guided Paths

Offer 35 days of psychoeducational content to teach the user how to utilize the tools in the app and help maintain motivation and interest. The lessons also provide background information about CBT and mindfulness. Each 5- to 10-min audio lesson is paired with an in-app activity. The audio content is presented either as a didactic session or a mock session between a client a therapist. The 35 days of content are divided into 4 paths: one which offers an introduction to each of the tools in the app, 2 which are sequential and offer a deeper focus on CBT, and one which focuses on building mindfulness skills.

#### Hope

On the basis of the principles of distress coping, the hope board is a user’s personal repository of inspirational quotes and images. It also allows the user to save their completed *challenges* to help them feel a sense of accomplishment and savor their wins.

#### Community

A peer-support community that is not moderated by professionals, but is rather a place where users can post their thoughts, challenges, and questions and receive support from others using the app. The community is anonymous but utilizes a flagging system to remove disruptive or inappropriate posts or users. Users are provided the rules and guidelines of the community when they first access the community.

#### Progress

Allows the user to review their mood ratings and completed activities. The tool graphs their mood ratings against health behaviors and other in-app activities so that users can identify patterns and triggers. There is also a *skills* tab that visually displays the user’s completed activities, separated by type.

#### Emergency Resources

Includes listing of crisis lines and resources for users in emergency situations.

### Measurement

#### Questionnaire Measures

Study participants were assessed at 3 time points: at baseline (pretreatment), 4 weeks later (ie, posttreatment; at the end of the intervention period), and 3 months after baseline/2 months postintervention (follow-up; only the Pacifica group completed the follow-up because the WL group was allowed to access the intervention after the 4-week assessment). At each assessment, participants were both emailed and notified via SMS that they had available assessments to complete. Assessments were completed via the secure Participant Portal described above (ie, not in the app itself).

The Depression Anxiety and Stress Scales-21 (DASS-21) [37] is a widely used freely available self-report measure of stress, anxiety, and depression. A shortened version of the DASS-42, its 21 items are measured on a Likert scale and yield 3 subscale scores: stress, anxiety, and depression. The DASS-21 consists of statements such as “I couldn’t seem to experience any positive feeling at all” with 4 response options ranging from “Did not apply to me at all” to “Applied to me very much or most of the time.” For each subscale, summed scores range from 0 to 21, with higher scores indicating greater symptoms. Its internal consistency and concurrent validity have been shown to be in the acceptable-to-excellent range [38].

The PHQ-8 [34] is an 8-item self-report measure of depression. It is identical to the popular PHQ-9 [39] measure but eliminates the question that queries suicidal ideation; it was selected given the all-virtual nature of the study. An example item from the PHQ-8 is “Little interest or pleasure in doing things,” with response options ranging from “Not at all” to “Nearly every day.” Scores on the PHQ-8 range from 0 to 24, and the measure has been shown to have good internal consistency and reliability [34]. Higher scores indicate greater symptoms.

The GAD-7 [33] is a widely used, freely available, 7-item self-report measure to address severity of Generalized Anxiety Disorder. An example item is “Feeling nervous, anxious, or on edge,” with response options ranging from “Not at all” to “Nearly every day.” Summed scores on the GAD-7 range from 0 to 21, with higher scores indicating greater symptoms. The GAD-7 has good reliability and validity [33].

The General Self-Efficacy Scale [40] is a 10-item self-report measure that asks participants about their beliefs regarding their ability to cope with stressors or challenges in their life. Example items include “I can always manage to solve difficult problems if I try hard enough,” with response options ranging from “Not at all true” to “Exactly true.” Scores on the General Self-Efficacy Scale range from 10 to 40, with higher scores indicating greater self-efficacy. The measure has been shown to be reliable and valid [41].

Overall, 2 composites were created from the 2 measures of depression (PHQ-8 and DASS-21 Depression Subscale) and the 2 measures of anxiety (GAD-7 and DASS-21 Anxiety Subscale) to generate more reliable and valid indices of symptoms. We standardized each measure at the pretreatment time point, then used the means and SDs calculated at pretreatment to standardize these measures at subsequent timepoints. After the scores were standardized, measures were averaged to create a single measure of each construct. These measures are referred to as *depression composite* and *anxiety composite* in the results section and have a mean of zero and a SD of one 1 pretreatment.

#### Process Measures

To better understand possible mediators of symptom improvement and app efficacy, we examined participants’ number of logins and number of completed activities as measures of app engagement. It should be noted that for the purposes of this study, *activities* include *thoughts*, *goals* (either setting or completing), *relax*, and *community* (writing a post). The engagement analysis did not include mood or health ratings, *hope* posts, or time spent reviewing content in the community forums because of the complexity involved in data extraction, cleaning, and formatting for these activities.

#### Moderator Measures

To examine whether baseline patient characteristics predicted treatment response to Pacifica versus WL, we tested demographic and clinical characteristics. Demographic features included age (treated as a continuous variable), gender (0=female, 1=male), income (8 levels from <$20K to >$200K treated as a continuous variable), marital status (0=not married, 1=married), education (0=did not complete 4-year college, 1=completed 4-year college), and race (0=other, 1=white). Clinical features included current (at screening) use of psychiatric medications (0=no, 1=yes), current (at screening) use of psychotherapy (0=no, 1=yes), use of other mental health apps (0=no, 1=yes), and whether they reported being diagnosed with depression (0=no, 1=yes) or anxiety (0=no, 1=yes).

### Statistical Analyses

#### Power Analysis

On the basis of a two-week pilot study among college undergraduates, we anticipated an effect size of .3 (Cohen *d*). We targeted 90% power to detect an effect to a significance level of .05 in a 2-group 1-tailed *t* test, which revealed a sample of 191 participants per group. The previous study had about 75% completion, so we targeted to randomize 250 participants per group. Though the use of pilot studies for estimating effect sizes is questionable (cf [42]), this pilot study was the most relevant data available on which to base our power analysis. The effect sizes found in this study are larger than those in the pilot study: between .4 and .54 for the key symptoms measures versus .3 in the pilot.

We were not able to meet our target of 191 completers because of greater-than-anticipated attrition: 101 WL participants and 79 active group participants completed the study. Given this number of completers, we were able to detect an effect size of .38 with 80% power given a significance level of .05 and a 2-group, 2-tailed *t* test.

#### Statistical Analysis

Data were analyzed using multilevel modeling (MLM) in Stata 15.0 [43]. MLM accounts for nesting of time points within subjects, which allows for examination of change within and between subjects across time (pretreatment, posttreatment, and follow-up) and by group (WL and Pacifica). MLM includes all participants with at least 1 measurement, and thus, it can be used to estimate intent-to-treat effects. Separate models were run for each of the 4 dependent variables (depression composite, anxiety composite, stress, and general self-efficacy). Statistical significance was defined as falling below a threshold of alpha=.05, as this is the standard threshold used in randomized clinical trials in psychology.

To examine the main effects of the Pacifica intervention compared with WL, time was modeled at level 1, with 1 segment specified between the pre- and posttreatment time points, and a second segment between the post and follow-up time points. This approach models typical trends in treatment studies where the greatest effects occur by posttreatment and changes level-off over follow-up and has been used in a number of previous studies [44,45,46,47]. Group (WL vs Pacifica) was modeled at level 2. Models included the main effects of the 2 time segments, the main effect of group, and the interaction between group and time. We examined the interaction between group and time for statistical significance. All models included random effects of the intercept and time, which were determined to be significant based on likelihood ratio tests. Between-group differences were assessed using the *margins* command in Stata by comparing the slopes from pre- to posttreatment and by comparing the predicted means from the model at the posttreatment time point. Effect sizes are calculated based on the method described by Feingold [48] that produces estimates analogous to Cohen *d* for growth curve models in randomized clinical trials. As the WL group received the intervention after the posttreatment time point, we were unable to compare the groups at follow-up. Thus, for the follow-up time point, the slope of change from posttreatment to follow-up was tested only for the Pacifica group.

To examine whether engagement with the app was related to treatment response, we tested whether overall engagement (number of app logins) and specific types of engagement (number of times using the goals, relaxation, community, and thoughts sections of the app), were associated with symptom change in the Pacifica group. Models included the main effect of engagement, the main effects of the 2 time segments, and the interactions between engagement and time. We examined the interactions between the engagement variables and each time segment for statistical significance. Thus, we examined whether engagement predicted change from pre- to posttreatment and from posttreatment to follow-up for each type of engagement and for each dependent variable.

To examine moderators of response to Pacifica versus WL, we tested whether demographic features (age, gender, income, marital status, educational level, and race) and clinical features (use of psychiatric medications, engagement in psychotherapy, use of other mental health apps, and previously diagnosed anxiety and depression) were associated with symptom change from pre- to posttreatment in the Pacifica group compared with the WL group. Models included the main effects of the moderator, the main effect of time, the main effect of group, all 2-way interactions, and the 3-way interaction. We examined the 3-way interaction between the moderator, group, and time for statistical significance. Significant 3-way interactions were followed up with tests of simple interaction effects of group by time at different levels of the moderator.

## Results

### Pretreatment Group Differences

Table 1 presents demographic data on the WL and Pacifica groups. Randomization was successful, and the 2 groups were very similar with regard to demographics; we found no significant differences between the groups (*p* s>.210). The sample was largely female (75%) and white (80%) and had at least some college education (91%).

### Study Attrition

Of the 500 participants randomized, 204 completed both pre- and posttreatment assessments. This represents 47% of WL participants and 35% of Pacifica participants. The difference in attrition between the groups was significant: *χ*_*1*_^2^ (n=500)=7.7; *P*=.006. We examined the baseline symptom ratings for the individuals who dropped out versus those who did not and found no differences (see Table 2). Further, no differences were found when these comparisons were stratified by group (*P*>.18).

**Table 1:** Descriptive data of sample.

Characteristic		Waitlist control group (n=247)	Active (n=253)	Chi-square (*df* ${3.1}	*P* value
Age (years), mean (SD)^a^		30.2 (10.8)	30.2 (10.9)	—^b^	.96
**Gender, n (%)**				**2.73 (4)**	**.60**
	Male	56 (23)	54 (21)	—	—
	Female	84 (74)	190 (75)	—	—
	Transgender man	4 (2)	2 (1)	—	—
	Nonconforming	3 (1)	6 (2)	—	—
**Race, n (%)**				**3.73 (5)**	**.59**
	Asian	11 (4)	10 (4)	—	—
	Black	20 (9)	28 (11)	—	—
	Hawaii/Pacific Islander	1 (0.5)	2 (1)	—	—
	Native American/Alaska Native	5 (2)	5 (2)	—	—
	White	208 (84)	202 (80)	—	—
	No reply	2 (1)	6 (2)	—	—
**Education level, n (%)**				**2.80 (5)**	**.73**
	Some high school	1 (0.5)	4 (2)	—	—
	High School	21 (9)	16 (6)	—	—
	Some college	81 (33)	86 (34)	—	—
	Associates	19 (8)	17 (7)	—	—
	Bachelor’s	80 (32)	84 (33)	—	—
	Master’s or higher	44 (17)	46 (18)	—	—
	No reply	1 (0.5)	0	—	—
**Marital Status, n (%)**				**1.50 (4)**	**.83**
	Never married	161 (65)	161 (64)	—	—
	Married	63 (26)	67 (26)	—	—
	Separated	3 (1)	6 (2)	—	—
	Divorced	19 (8)	17 (7)	—	—
	Widowed	1 (0.5)	2 (1)	—	—
**Income level in USD, n (%)**				**9.62 (7)**	**.21**
	Under 20K	101 (41)	97 (38)	—	—
	20-35k	50 (20)	39 (15)	—	—
	35-50k	38 (15)	38 (15)	—	—
	50-75k	25 (10)	46 (18)	—	—
	75-100k	17 (7)	20 (8)	—	—
	100-150k	9 (4)	9 (4)	—	—
	150-200k	3 (1)	3 (1)	—	—
	Over 200k	4 (2)	1 (0)	—	—
**Using other apps for mental health**				**0.09 (1)**	**.77**
	Yes	52 (21)	56 (22)	—	—
	No	195 (79)	197 (78)	—	—
**Currently in therapy**				**0.47 (1)**	**.49**
	Yes	57 (23)	52 (21)	—	—
	No	190 (77)	201 (79)	—	—
**Currently taking psychiatric medication**				**1.61 (2)**	**.45**
	Yes	91 (37)	101 (40)	—	—
	No	155 (6)	151 (60)	—	—
	No response	1 (0)	1 (0)	—	—
**Previous diagnosis of depression**				**0.13 (1)**	**.72**
	Yes	184	192	—	—
	No	63	54	—	—
**Previous diagnosis of anxiety**				**0.13 (1)**	**.72**
	Yes	205	213	—	—
	No	42	40	—	—

^a^*t*_498_=0.05.

^b^Not applicable.

**Table 2:** Mean values on baseline outcomes for participants who dropped versus completed the posttreatment assessment.

Measure	Drop	Complete	*t* (*df*)	P value
Depression composite	−0.01	0.01	0.31 (498)	.76
Anxiety composite	0.04	−0.05	−1.16 (498)	.25
Depression Anxiety and Stress Scales-21 stress	8.77	8.55	−0.78 (498)	.44
General self-efficacy	26.97	26.75	−0.57 (498)	.57

### Key Outcome: Symptom Change

We first examined whether, compared with individuals in the WL group, individuals assigned to use Pacifica experienced greater improvement in anxiety, depression, stress, and self-efficacy. Our intent-to-treat analysis revealed significant group × time interactions for each of the outcome measures (depression composite, anxiety composite, DASS-21 stress, and self-efficacy) such that change in the Pacifica group was greater than change in the WL group. These results were unchanged when individual measures of depression and anxiety were examined separately. See Multimedia Appendix 1 for means and SEs of the individual depression and anxiety measures. In addition, to assess whether treatment dropout affected these results, we analyzed additional models that included the group × dropout interaction and main effects of group and dropout, and the results were unchanged. Table 3 provides parameter estimates for the group × time interaction from pre to posttreatment, 95% CIs, significance levels, and effect sizes for each outcome measure.

**Figure 2:**
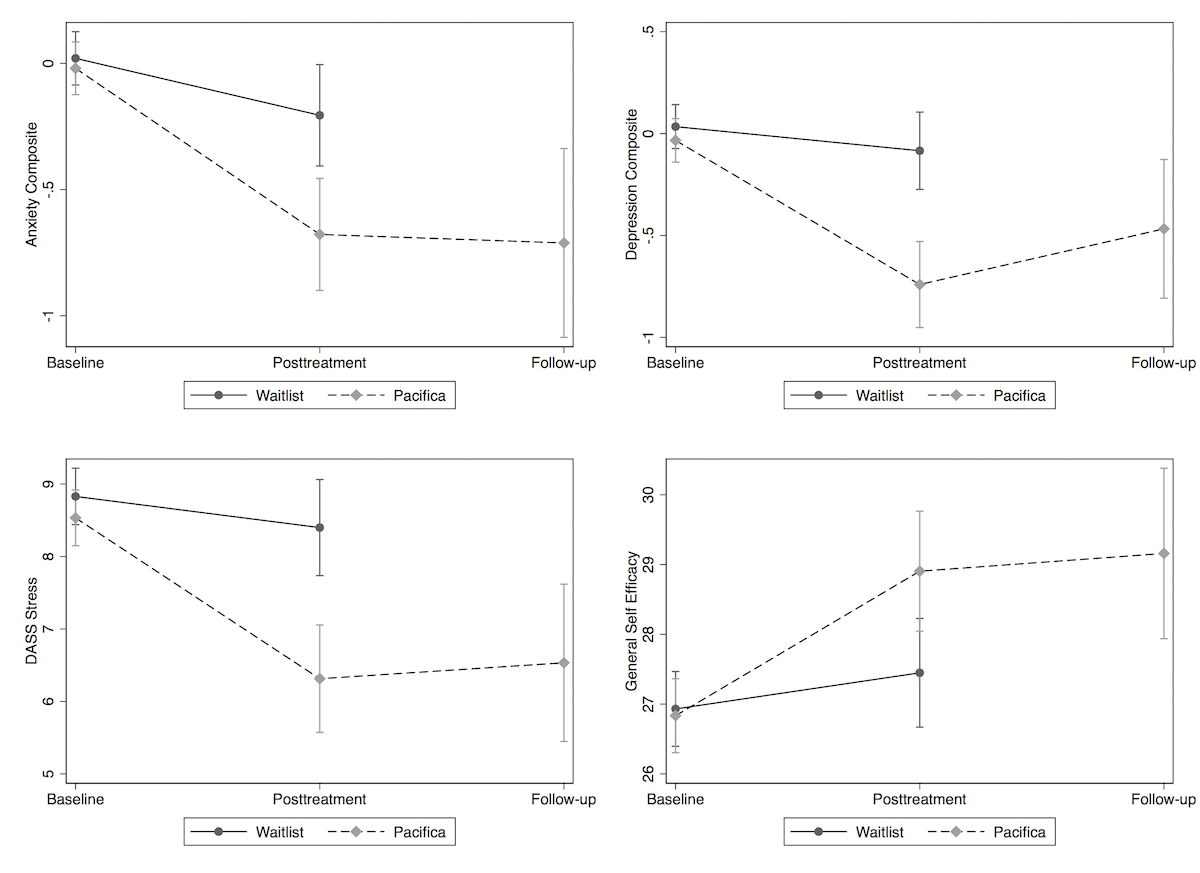
Key outcome measures by group and time. DASS: Depression Anxiety Stress Scales.

We additionally examined the simple effect of time within groups. Table 4 shows that the Pacifica group experienced significant change from pre to post for each variable (decreases for depression, anxiety, and stress and an increase for self-efficacy). Anxiety level in the WL group significantly decreased from pre to post. For the Pacifica group, from post to follow-up, there were no significant changes in any outcome. There were significant group differences at post for each measure (see Figure 2). The WL group was higher than the Pacifica group for depression symptoms (0.66; CI 0.37 to 0.94; *P*<.001), anxiety symptoms (0.47; CI 0.17 to 0.77; *P*=.002), and DASS-21 stress (2.09; CI 1.09 to 3.08; *P*<.001), and lower on self-efficacy (−1.46; CI −2.62 to −0.30; *P*=.014).

**Table 3:** Group × time interaction.

Measure	Beta	95% CI	*P* value	Effect size (*d*)
Depression composite	−0.59	−0.86 to -.03	<.001	0.54
Anxiety composite	−0.43	−0.71 to −0.15	.003	0.40
Depression Anxiety and Stress Scales-21 stress	−1.79	−2.74 to −0.84	<.001	0.46
General self-efficacy	1.55	0.53 to 2.58	.003	0.34

**Table 4:** Score change on key measures across time by group.

Measure			Change across time	95% CI	*P* value
**Depression composite**					
	**Pre versus post**				
		Control	−0.12	−0.30 to 0.06	.21
		Pacifica	−0.71	−0.91 to 0.50	<.001
	**Post versus Follow-up**	
		Pacifica	0.27	−0.10 to 0.64	.149
**Anxiety composite**					
	**Pre versus post**				
		Control	−0.23	−0.41 to −0.04	.018
		Pacifica	−0.66	−0.87 to −0.45	<.001
	**Post versus Follow-up**	
		Pacifica	−0.03	-0.44 to 0.37	.87
**Depression Anxiety and Stress Scales-21 stress**					
	**Pre versus post**				
		Control	−0.43	−1.06 to 0.20	.18
		Pacifica	−2.22	−2.93 to −1.51	<.001
	**Post versus Follow-up**	
		Pacifica	0.22	−0.97 to 1.41	.72
**General self-efficacy**					
	**Pre versus post**				
		Control	0.52	−0.16 to 1.2	.13
		Pacifica	2.10	1.30 to 2.84	<.001
	**Post versus Follow-up**	
		Pacifica	0.25	−1.02 to 1.53	.697

### Clinically Significant Change

Participants were classified as having achieved clinically significant change (CSC) on the PHQ-8 and GAD-7 as outlined by Jacobson and Truax [49]. The individual scales were used for the CSC analysis because test-retest reliability metrics were needed to calculate the reliable change index. Rates of CSC at post for PHQ-8 were as follows: Pacifica, 41.8% (n=33/79); WL, 16.8% (n=17/101); *χ*_*1*_^2^ (N=180)=13.74; *P*<.001. Rates of CSC at post for GAD-7 were as follows: Pacifica, 39.2% (n=31/79); WL, 24.2% (n=24/99); *χ*_*1*_^2^ (N=178)=4.63; *P*=.031. The rate of CSC at follow-up for the Pacifica group was 35.4% for the PHQ-8 and 48.9% for the GAD-7.

### Engagement Effects

The median number of logins in the Pacifica group during the 30-day intervention was 19, with a range of 1 to 286. We did not find any significant interactions for group by time by *overall* engagement with the app (defined by total number of logins) for depression composite (*P*>.21), anxiety composite (*P*>.34), stress (*P*>.11), or self-efficacy (*P*>.55).

**Figure 3:**
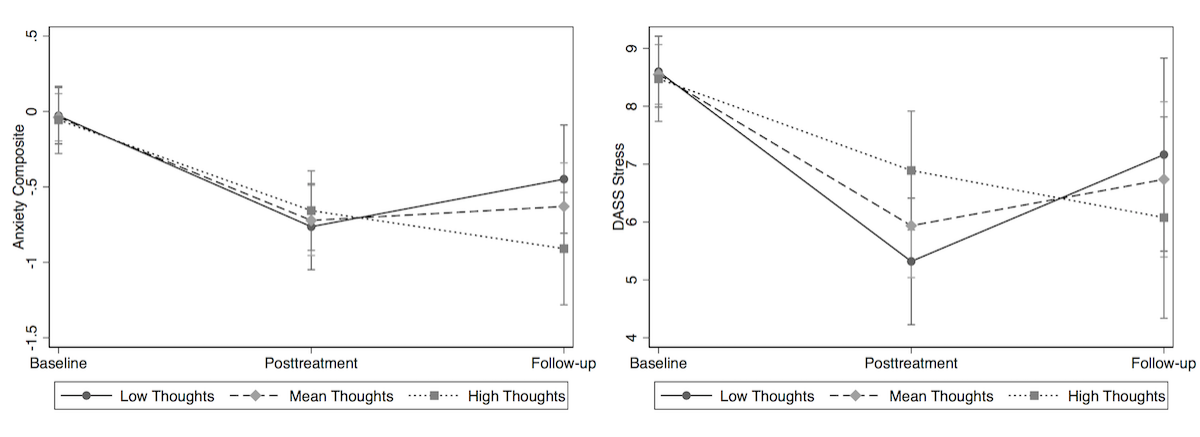
Interaction between use of "Thoughts" tool and anxiety and stress outcomes. DASS: Depression Anxiety Stress Scales.

However, for tests examining unique activities in the app, we found that the use of thought record tools was significantly associated with symptom improvement for 2 of the outcome measures: anxiety composite and stress (see Figure 3). Specifically, for the anxiety composite, greater number of thought records completed during treatment was associated with greater anxiety reduction from posttreatment to follow-up (beta *=*-.10; CI −0.19 to −0.02; *P=*.019), although not from pretreatment to posttreatment (*P=*.406) *.* Number of thought records completed was not significantly associated with baseline anxiety in the MLM (*P*=.834). For DASS-21 stress, a greater number of thought records completed during treatment was associated with less stress reduction from pre- to posttreatment (beta *=* −.30; CI −.50 to −.11; *P*<.01), but greater stress reduction from posttreatment to follow-up (beta *=*-.47; CI −.87 to −.08; *P*<.05). Number of thought records completed was not significantly associated with baseline stress in the MLM (*P*=.772). Descriptively, individuals who completed relatively more thought records showed a delayed positive effect in terms of anxiety and stress reduction. Use of the thought record tool was not significantly associated with change in depression (*P*>.13) or general self-efficacy (*P*>.48). The use of the goals, relax, and community tools was not associated with change in anxiety (*P*>.18), depression (*P*>.13), stress (*P*>.29), or general self-efficacy (*P*>.26).

### Moderator Analysis

#### Demographics

We examined the 3-way interaction between group, time, and demographic responses to examine whether any of these demographic traits moderated treatment response.

Age, gender, income, marital status, educational level, and race did not significantly moderate the effect of group on symptom improvement. See Table 5 for details.

#### Clinical Features

**Figure 4:**
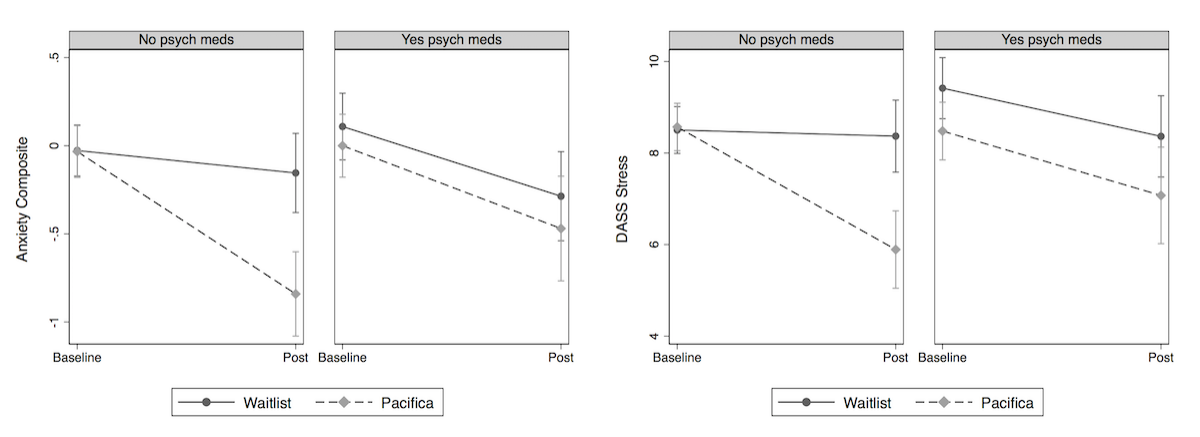
Moderation of group by time effect by participant self-reported psychiatric medication use. DASS: Depression Anxiety Stress Scales.

We also conducted moderator analyses for each of the clinical characteristics listed in Table 1. We found only 1 characteristic that significantly interacted with group and time to predict outcome measures: presence/absence of concomitant psychiatric medications at baseline. As shown in Figure 4, the presence of psychiatric medications moderated the group effect such that the benefits of Pacifica compared with WL were significantly greater for people who were not taking psychiatric medications than for people who were taking medications. This moderation of the group × time interaction only reached significance for the anxiety composite and DASS-21 stress measures.

For the anxiety composite, tests of simple effects showed that for participants not taking psychiatric medications, those assigned to Pacifica had a significantly greater reduction in anxiety than those assigned to WL (*slope difference*=−.68, CI −1.01 to −0.35; *P*<.001). However, for those taking psychiatric medications, no difference in symptom reduction emerged between Pacifica and WL (*P*=.771). A similar pattern emerged for stress. For participants not taking psychiatric medications, those assigned to Pacifica had a significantly greater reduction in stress than those assigned to WL (*slope difference*=−2.54; CI −3.70 to −1.39]; *P*<.001). However, for those taking medications, no difference in symptom reduction emerged between Pacifica and WL (*P*=.615).

**Table 5:** Group × time × moderator.

Variable	Depression, beta (95% CI)	Anxiety, beta (95% CI)	DASS-21 stress, beta (95% CI)	Self-efficacy, beta (95% CI)
Age (years)	−.01 (−0.03 to 0.02)	−.01 (−0.03 to 0.02)	.01 (−0.08 to 0.09)	−.03 (−0.13 to 0.06)
Gender	−.16 (−0.81 to 0.49)	−.08 (−0.73 to 0.57]	−1.72 (−4.02 to 0.57)	−.90 (−3.40 to 1.61)
White	−.22 (−0.93 to 0.48)	−.40 (−1.11 to 0.30)	.18 (−2.29 to 2.65)	.50 (−2.20 to 3.20)
College educated	.15 (−0.36 to 0.65)	.48 (−0.03 to 0.99)	.60 (−1.20 to 2.39)	1.35 (−0.63 to 3.34)
Married	−.25 (−0.81 to 0.32)	−.45 (−1.02 to 0.12)	−1.85 (−3.85 to 0.15)	1.30 (−0.88 to 3.49)
Income level	.04 (−0.12 to 0.19)	.09 (−0.07 to 0.25)	.27 (−0.29 to 0.84)	−.08 (−0.70 to 0.53)
Using other apps	.01 (−0.59 to 0.62)	−.18 (−0.79 to 0.42)	−.31 (−2.44 to 1.81)	.67 (−1.68 to 3.02)
Receiving psychotherapy	.17 (−0.46 to 0.81)	.44 (−0.20 to 1.08)	.52 (−1.76 to 2.80)	−1.81 (−4.39 to 0.78)
Taking psychiatric medication	.47 (−0.04 to 0.98)	.61 (0.09 to 1.12)^a^	2.19 (0.39 to 3.99)^a^	.18 (−1.84 to 2.20)
Diagnosed with depression	−.27 (−0.84 to 0.30)	.13 (−0.44 to 0.70)	.30 (−1.72 to 2.32)	.80 (−1.40 to 3.00)
Diagnosed with anxiety	.21 (−0.51 to 0.93)	−.02 (−0.75 to 0.72)	.17 (−2.38 to 2.73)	−1.18 (−4.00 to 1.64)

^a^Effects are significant at *P*<.05.

## Discussion

This study is the first published RCT of Pacifica, a widely available, popular smartphone app–based intervention for self-help of mild-to-moderate stress, anxiety, and depression. The tools implemented in the intervention are based on the integration of CBT, mindfulness, and mood and health tracking. Results indicated that this intervention is effective compared with a waitlist control at 1 month in reducing self-reported symptoms of depression, anxiety, and stress and increasing feelings of self-efficacy. Between-group effect sizes were in the small-to-medium range. In addition, treatment gains were maintained for 2 months following the end of the 1-month intervention period, especially for anxiety symptoms. These results are particularly noteworthy given the very light touch nature of the study, short duration of the intervention period, and the real-world sample of individuals.

App usage was self-guided, and participants were not instructed to use the app with any specific level of frequency, so rather than consider adherence, we explored whether amount of usage (ie, number of completed activities) was associated with symptom improvement. We did not find any association with overall usage, possibly because looking at the total number of app logins is a simplistic way of quantifying engagement. However, we did find that individuals who completed relatively more thought records demonstrated delayed improvement, which is noteworthy. Though there have been challenges in the literature to the importance of cognitive restructuring in CBT [50], this study suggests that, at least for technology-enabled CBT, thought records were helpful. Subjectively, completing thought records is a more time-intensive and cognitively demanding activity than health tracking or meditation and increased use of these activities may be a marker of greater commitment to treatment or improvement. We should note, though, that in this study thought records included both basic journaling prompts and more complex reframing activities. However, this study was unfortunately underpowered to examine the effects of different types of thought tools. The apparent delayed positive benefit of completing relatively more thought activities is consistent with the common perception that although examining and challenging negative thoughts is difficult in the short term, it can result in more long-lasting benefits. In addition, there are data suggesting that CBT is effective at preventing relapse to anxiety and depression [51,52], but further research is needed to more completely understand these effects, particularly in computerized CBT.

Demographic characteristics among participants were not related to outcomes, which is consistent with the mixed findings across previous studies examining traditional (ie, not online delivered) CBT [53-55] as well as internet-based CBT [13,25,56-60]. This suggests that app-based CBT can have effects across diverse populations rather than being relevant only for a specific group. However, we found differential effects between participants who were and were not taking psychiatric medications at baseline. Though not significant for measures of depression or self-efficacy, for the anxiety composite and DASS-21 stress measures, the presence of medication at baseline diminished the difference between the groups, that is, both the WL and app groups improved. Inspection of the means reveals not that the presence of medications markedly reduced the effectiveness of the app, but rather that there was little added benefit of the app above and beyond the medication.

Although this finding is inconsistent with some data that combined treatments perform better than therapy alone [61], it is consistent with literature that has found minimal benefit of combining medication with psychotherapy [62]. Certain medications such as benzodiazepines may interfere with behavioral therapy [63]. To be sure, though, a guided self-help tool such as Pacifica is not psychotherapy—nor does it purport to be. The personal relationship established with a practitioner may be what catalyzes treatment and nudges it above medication alone; blended models that incorporate both self-help solutions with human coaching have been found to be superior to self-help alone [64]. The drawback of these models is their ability to scale. Stepped care models that triage services and match the level of in-person support with client need are likely the way of the future [65]. Such models combine the rapport and human connection of a human therapist or coach with the ubiquity and outside-of-the-clinic support of a mobile app. The question of whether combining mobile interventions with pharmacotherapy is effective is particularly relevant given the fact that a majority of individuals receive their psychotropic medications from primary care providers [66] who typically only see them intermittently; digital tools could potentially provide additional support between appointments.

This project represents a step forward in that it is a real-world evaluation of a popular, commercially available technology-enabled intervention. However, there are several limitations that should be noted. First, the study was conducted among a convenience sample that was rather homogeneous in its demographic makeup. The participants were largely college-educated white females. Previous research has found that women are overrepresented in Web-based research studies [67]. Although this sample may represent the individuals who are most likely to utilize technology-enabled mental health interventions, this homogeneity may limit the degree to which the outcomes can be generalized. Owing to the fact that no demographic information is collected from users upon sign-up, we cannot assess how representative this sample is of all of the individuals who utilize Pacifica.

Although the participant pool was homogeneous from a demographic perspective, the study was conducted in a sample that may have been heterogeneous with respect to diagnosis. We did not conduct diagnostic interviews and only have participants’ responses to symptom rating questionnaires. In the real world, many individuals seeking Web-based tools to manage their mental health are doing so outside of the context of the therapy clinic and may not have formal diagnoses. It is noteworthy that our sample had relatively low levels of anxiety and depression. This further highlights the fact that individuals who seek digital tools may be on the mild end of the spectrum and that these tools may need to be used in conjunction with therapy to be effective for more severely affected individuals. Future research should examine whether these data can be replicated in a more diverse sample to consider whether efficacy varies based on demographics. It would also be important to verify the app’s efficacy among individuals who have been formally diagnosed versus those who self-selected and were simply screened using self-reported measures. Furthermore, analyses of engagement with the app were exploratory, and significant findings should be replicated in future studies.

Another limitation relates to the finding regarding the moderation of the effects by the use of psychotropic medications. Although this finding is interesting, because we had minimal information about participants’ use of medications (ie, no data on the types, dosage, or any change to medication across the course of the study), this should be examined further in future work.

This study provides encouraging findings around the ability of popular, commercially available, guided self-help tools to empower individuals to manage their symptoms of stress, anxiety, and depression and increase their self-efficacy. This study also serves as a proof point for an all-virtual study. Given the limitations of existing health care systems and the obstacles to care that exist for individuals coping with mental health conditions, mobile apps and technology-enabled interventions can play an important role in expanding access and serving as an adjunct to in-person treatment. Future research should continue to clarify the best application of these tools and how they can be better integrated into existing workflows and care delivery systems.

## Appendix

Multimedia Appendix 1 Predicted means and standard errors for individual depression (Patient Health Questionnaire, Depression Anxiety Stress Scales-Depression) and anxiety (Generalized Anxiety Disorder 7-item, Depression Anxiety Stress Scales-Anxiety) scales.

Multimedia Appendix 2 CONSORT-EHEALTH checklist (V 1.6.1).

## Abbreviations

CBT: cognitive behavioral therapy
CSC: clinically significant change
DASS-21: Depression Anxiety Stress Scales-21
EAP: employee assistance program
GAD-7: Generalized Anxiety Disorder 7-item
MLM: multilevel modeling
PHQ-8: Patient Health Questionnaire 8-item
RCT: randomized controlled trial
SMS: short messaging service
