# Supplementary material for: Guided Self-Help Works: Randomized Waitlist Controlled Trial of Pacifica, a Mobile App Integrating Cognitive Behavioral Therapy and Mindfulness for Stress, Anxiety, and Depression
Source: J Med Internet Res. 2019 Jun 8;21(6):e12556. doi: 10.2196/12556 (PMC6592477; doi:10.2196/12556)
Supplement: Multimedia Appendix 1 [file jmir_v21i6e12556_app1.pdf]

Supplementary Table 1.

	Pre		Post		Follow-Up
	**Pacifica**	**WL**	**Pacifica**	**WL**	**Pacifica**
PHQ-8	9.7 (.2)	10.0 (.2)	7.4 (.4)	9.5 (.3)	8.2 (.6)
DASS-Depression	7.6 (.3)	7.8 (.3)	5.4 (.4)	7.7 (.6)	6.1 (.6)
GAD-7	9.7 (.2)	9.6 (.2)	7.4 (.4)	8.8 (.4)	6.8 (.7)
DASS-Anxiety	5.5 (.2)	5.9 (.2)	3.8 (.3)	5.3 (.3)	4.3 (.5)

Note. PHQ-8 = Patient Health Questionnaire; DASS = Depression, Anxiety, and Stress Scale; GAD-7 = Generalized Anxiety Disorder 7-item Scale
