# Supplementary material for: Guided Self-Help Works: Randomized Waitlist Controlled Trial of Pacifica, a Mobile App Integrating Cognitive Behavioral Therapy and Mindfulness for Stress, Anxiety, and Depression
Source: J Med Internet Res. 2019 Jun 8;21(6):e12556. doi: 10.2196/12556 (PMC6592477; doi:10.2196/12556)
Supplement: Multimedia Appendix 2 [file jmir_v21i5e12556_fig.pdf]

POND5

Proof of purchase

A license to use the following media was purchased under Pond5's Content License Agreement, a copy of which is available for review at https://www.pond5.com/legal/license. The Pond5 license authorizes the licensee to use the media in the licensee's own commercial or non-commercial production and to copy, broadcast, distribute, display, perform and monetize the production or work in any medium - including posting and monetization on YouTube - on the terms and conditions outlined therein.

	MEDIA TITLE		MEDIA TYPE	CONTRIBUTOR	DATE PURCHASED	LICENSE
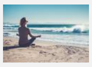	young woman meditating on the beach	36944784	Photos	amoklv	2015-04-11	Royalty Free License v20150107

Pond5 Inc. 251 Park Ave S, 7th Floor - New York, NY 10010, USA - US Tax ID: 26-1575318
